# Newly Synthesized Lignin Microparticles as Bioinspired Oral Drug-Delivery Vehicles: Flavonoid-Carrier Potential and In Vitro Radical-Scavenging Activity

**DOI:** 10.3390/pharmaceutics15041067

**Published:** 2023-03-26

**Authors:** Donika Ivanova, Monika Toneva, Evgeni Simeonov, Biliana Nikolova, Severina Semkova, Georgi Antov, Zvezdelina Yaneva

**Affiliations:** 1 Department of Pharmacology, Animal Physiology, Biochemistry and Chemistry, Faculty of Veterinary Medicine, Trakia University, Students Campus, 6000 Stara Zagora, Bulgaria; 2 Department of Chemical Engineering, Faculty of Chemical and System Engineering, University of Chemical Technology and Metallurgy, 8 Kliment Ohridski Blvd., 1756 Sofia, Bulgaria; 3 Institute of Biophysics and Biomedical Engineering, Bulgarian Academy of Sciences, Acad. Georgi Bonchev Str., Block 21, 1113 Sofia, Bulgaria; 4 Institute of Plant Physiology and Genetics, Bulgarian Academy of Sciences, Acad. G. Bonchev Str., Bldg. 21, 1113 Sofia, Bulgaria

**Keywords:** lignin microparticles, morin, in vitro release, antioxidant activity

## Abstract

The aim of the present study was to synthesize lignin microparticles, to evaluate their physicochemical, spectral, morphological and structural characteristics, to examine their encapsulation and in vitro release potential and behaviour towards the flavonoid morin in simulated physiological medium and to assess the in vitro radical-scavenging potential of the morin-loaded lignin microcarrier systems. The physicochemical, structural and morphological characteristics of alkali lignin, lignin particles (LP) and morin-encapsulated lignin microparticles (LMP) were determined based on particle size distribution, SEM, UV/Vis spectrophotometric, FTIR and potentiometric titration analyses. The encapsulation efficiency of LMP was 98.1%. The FTIR analyses proved that morin was successfully encapsulated in the LP without unexpected chemical reactions between the flavonoid and the heteropolymer. The in vitro release performance of the microcarrier system was successfully mathematically described by Korsmeyer–Peppas and the sigmoidal models outlining the general role of diffusion during the initial stages of the in vitro release process in simulated gastric fluid (SGF), and the predominant contribution of biopolymer relaxation and erosion was determined in simulated intestinal medium (SIF). The higher radical-scavenging potential of LMP, as compared to that of LP, was proven via DPPH and ABTS assays. The synthesis of lignin microcarriers not only provides a facile approach for the utilization of the heteropolymer but also determines its potential for the design of drug-delivery matrices.

## 1. Introduction

Although, as the most abundant aromatic heteropolymer of natural origin, lignin has recently started to attract the interest of researchers and industries worldwide, it is still underutilized and its potential is underestimated.

The lignin molecular structure is a cross-linked network comprised of three basic monolignols acting as the major building blocks. These units are *p*-hydroxyphenyl, derivatives of *p*-coumaryl alcohol; guaiacyl, derivatives of coniferyl alcohol; and syringyl, derivatives of sinapyl alcohol. The lignin mono units are bonded together via phenylcoumaran, ether, diarylpropane, biphenyl bonds. In addition, the heteropolymer macromolecules contain a variety of functional groups, such as methoxy (-OCH_3_), phenolic hydroxyl (Ar-OH), aliphatic hydroxyl (R-OH), benzyl alcohol, non-cyclic benzyl ether, carbonyl and carboxyl groups [1,2,3,4].

Investigations of the antioxidant, antibacterial, antiproliferative and anti-ultraviolet radiation properties of lignin have drawn scientific attention, due to the hetero biopolymer potential for high-value-added applications in biomedicine, pharmacy, food industry, cosmetic and health product fields, as well as new for the generation of 3D printing precursor materials [5,6,7].

Provoked by the latter, nowadays, the design of nano-/micro-formulations based on lignin has gained interest for application as drug delivery systems due to their potential as entrapping and encapsulation vehicles of pesticides, drugs and enzymes, as surfactants, as reinforcing agents in nanocomposites, sorbents for heavy metal ions, organic priority pollutants [8] and other environmental contaminants and for antibacterial and antioxidant applications [9,10]. The literature reports diverse approaches for the production of micro-/nano-scale lignin formulations: solvent/pH shifting [11], acid-catalysed precipitation; flash precipitation [12], dialysis, supercritical antisolvent process, W/O microemulsion methods [13], spray drying, polymerization, ice-segregation, aerosol processing, mechanical processing [9], interfacial crosslinking, self-assembly via emulsion-solvent diffusion, emulsion solvent evaporation, antisolvent precipitation, sonochemical synthesis [14,15,16,17]. Spherical lignin micro-/nanoparticles exhibit superior properties as compared to powdered lignins, such as excellent surface activity, high UV shielding, significant oxidation resistance, and satisfactory antimicrobial potential, etc. [14]. According to the study of Gomide et al. (2020), the ultrasonic treatment of lignin particles leads to a reduction in their size, which provokes a decrease in the zeta potential and lower probability of agglomeration and increases the thermal stability and the antioxidant potential of the microparticles [9]. Boarino et al. (2022) explored the use of polylactic acid-grafted lignin nanoparticles for enhancement of the antioxidant activity and the mechanical and UV-barrier properties of polylactic acid food packaging films [18]. Porphyrin-encapsulated acetylated lignin nanoparticles exhibited stable behaviour within a wide pH range, preserving its photophysical properties, and demonstrated a photodynamic eradication effect on Gram-positive bacterial strains [19]. Yearla and Padmasree (2016) synthesized dioxane lignin nanoparticles and alkali lignin nanoparticles via a nanoprecipitation method and reported their improved antioxidant activity and UV protective properties, as compared to those of the parent polymers [20].

The three-dimensional polymer structure of lignin, comprising of both hydrophobic and hydrophilic groups, determines its applicability and efficiency in tissue engineering and in drug/gene transport, as well as its compatibility with biological media [16,17]—facts that explicate the constantly increasing scientific interest towards the biopolymer as a potential source for the synthesis of controlled delivery carriers [14].

Modern scientific literature provides evidence that in vitro systems for drug delivery based on micro-/nano biopolymer carriers ensure the integration of bioactive molecules and protect them from degradation along their physiological distribution pathways, thus meeting the challenging objective of their delivery to a specific area of therapeutic action [14].

Diverse scientific studies report in vitro and in vivo antioxidant, anti-inflammatory, antibacterial and anti-proliferative activities of the natural bioflavonoid morin. Though various aspects of the properties and pharmacokinetics of the bioactive plant derived substance have been established, among the major limitations for its efficient therapeutic applicability are the high photosensitivity, low solubility in water and poor oral bioavailability of morin [21,22,23,24]. The latter justified its application as a model bioactive substance in the current investigations.

Literature surveys outline quite recent scientific studies with still a limited number of reports on the encapsulation and release behaviour of natural polyphenols in/from lignin-based micro-/nano-formulations and in-depth characterizations of the carrier physicochemical properties and bioactivities so far [25,26,27]. In the context of the delineated scientific gaps, the present study was designed to synthesize lignin microparticles, to evaluate their physicochemical, spectral, morphological and structural characteristics, to examine their encapsulation and in vitro release potential towards morin in simulated physiological medium and to assess the in vitro radical-scavenging potential of the morin-loaded lignin microcarrier systems. Moreover, the application of volatile and flammable solvents, such as tetrahydrofuran, acetone, methanol and even DMSO at a high concentration, etc., which are typically used to produce lignin-based micro-/nano-particles, was avoided in the present study and envisaged as the novelty of the synthesis methodology. In the cases when such inherently hazardous chemicals are applied, they might remain in the particles, limiting their application in the biomedical field [25]. The synthesis method applied in the current study is based on the potential of the displacement of the green solvent of alkali lignin—water, with a negligible quantity of inorganic acid. Thus, the use of alternative solvents and non-toxic compounds as surfactants presents a greener option that avoids safety issues.

## 2. Materials and Methods

### 2.1. Chemicals

The reagents applied in the present study included the following: lignin (alkali, CAS No: 8068-05-1), morin (C_15_H_10_O_7_, purum, CAS: 654055-01-3), Tween 80 (CAS No: 9005-65-6, for synthesis), DPPH (2,2-Diphenyl-1-(2,4,6-trinitrophenyl)hydrazyl, C_18_H_12_N_5_O_6_, CAS No.:1898-66-4), ABTS (ABTS™ chromophore, diammonium salt, C_18_H_18_N_4_O_6_S_4_·(NH_3_)_2_, CAS No: 30931-67-0), ethanol (EtOH, C_2_H_5_OH, p.a. ≥ 99.8%), NaOH (p.a., HPLC), HNO_3_ (p.a., HPLC), HCl (ACS reagent, 37%), phosphate-buffered saline (PBS, P-3813), K_2_S_2_O_8_ {CAS No. 7727-21-1ACS reagent, ≥99.0%), Folin–Ciocalteu’s phenol reagent, Na_2_CO_3_ (powder, ≥99.5%, ACS reagent) and ascorbic acid (C_6_H_8_O_6_, CAS No: 50-81-7), supplied by Sigma-Aldrich (St. Louis, MA, USA).

Concerning safety issues with regards to alkali lignin, it has to be stated that although according to the European Chemicals Agency, alkali lignin (CAS No. 8068-05-1) falls under the hazard classification as it may cause eye irritation, respiratory irritation or allergic skin reaction, it is not classified as a toxic substance [28]. In addition, in the Safety Data Sheet according to Regulation (EC) No. 1907/2006, provided by Sigma-Aldrich (St. Louis, MA, USA), alkali lignin is “Not a hazardous substance or mixture according to Regulation (EC) No. 1272/2008” and “This substance/mixture contains no components considered to be either persistent, bioaccumulative and toxic (PBT), or very persistent and very bioaccumulative (vPvB) at levels of 0.1% or higher.” [29].

### 2.2. Synthesis of Lignin and Morin-Loaded Lignin Microparticles

Lignin microparticles were synthesized using a solvent–antisolvent precipitation procedure modified by us. Briefly, 1% Tween 80 was added to 25 mL aqueous alkali lignin solution at a concentration of 50 g/L. The mixture was agitated on a magnetic stirrer followed by the drop-by-drop addition of 2N HNO_3_. The obtained suspension was centrifuged for 30 min at 15.000 × *g* on an ultracentrifuge Hermle Z 326 K (HERMLE Labortechnik GmbH, Wehingen, Germany) at 10 °C. The supernatant was removed and the microparticles were rinsed with Milli-Q water. The rinsing/ultracentrifugation procedures were repeated three times after which the particles were homogenized for 4 min at an intensity of 97% on an ultrasound homogenizer Bandelin Sonopuls HD 2070 (BANDELIN Electronic GmbH & Co. KG, Berlin, Germany) and lyophilized at a temperature of –64 °C on a Biobase freeze dryer (Biobase Bioindustry Ltd., Jinan, China).

The synthesis of the morin-encapsulated lignin microparticles followed a similar procedure. The flavonoid (37.5 mg) was added to the mixture of alkali lignin/Tween 80 before the addition of the precipitant 2N HNO_3_.

The encapsulation efficiency (*EE*, %) of the natural flavonoid was calculated based on the formula:

(1) $$EE,\;\%=\frac{total\;bioactive\;substance\;added-free\;non-entrapped\;biactive\;substance\;}{total\;bioactive\;substance\;added}\times 100$$

### 2.3. Characterization of LP and LMP

Particle size and particle size distribution: The particles size and particle size distribution of the samples was assessed using an EVE automatic-cell counter (NanoEnTek, Seoul, Korea) with the option for bead counts via the addition of 1 µL of a microparticle suspension in Milli-Q water on an EVE™ counting slide required for the operation. The automatic cell counter presented the number of particles in 1 µL, as well as their distribution by size.

Content of acidic groups: The content of the acid groups (meq/g) in alkali lignin and LP was determined via the potentiometric titration of 0.1 M HCl solution of lignin and the suspension of microparticles containing known amounts of the analytes with 0.1 M NaOH as a titrant. The solution pH was measured on a Consort C931 pH-meter (Consort, Belgium) [2]. The contents of the acidic groups were calculated on the basis of the zero and first-order differential titration curves and the corresponding equivalent points.

Total phenolic content (TPC): The total phenolic content of alkali lignin, LP and LMP was determined via the Folin–Ciocalteu colorimetric method, as described by Singleton et al. (1999) with some modifications [30]. Briefly, 200 µL of sample—aqueous suspension/solution—was mixed with 600 µL Milli-Q water and 200 µL Folin–Ciocalteu reagent (1:1, *v*/*v*). After 5 min, 1.0 mL of 8% Na_2_CO_3_ and 1.00 mL Milli-Q water were subsequently added to the mixture and incubated at 40 °C for 30 min in a water bath with intermittent agitation. Afterwards, the absorbance was measured on a DR 5000 UV/Vis spectrophotometer (Hach-Lange, Düsseldorf, Germany) at 760 nm against a blank without extract. The outcome data were expressed as mg of gallic acid equivalents in milligrams per gram (mg GAE/g) of dry sample. All experiments were carried out in triplicate, and the average absorbance values obtained at different concentrations of gallic acid were used to plot the calibration curve. The total phenolic contents in all the samples were calculated by Equation (2):

(2) $$TOC=\frac{C\cdot V}{m}$$

where *C* is the concentration equivalent to that of gallic acid obtained from the calibration curve (mg/L), *V*—volume of the solvent (L), and *m*—mass of the dry sample (g).

SEM analyses: The morphology of the particles was characterized using a scanning electron microscope (SEM) JEOL 5510 (Jeol Ltd., Tokyo, Japan).

FTIR analyses. The FTIR spectra of the pure substances morin, lignin and the microparticles were obtained with the potassium bromide (KBr) disc technique in the range 400–4000 cm^−1^ using a TENSOR 37 Bruker FTIR spectrometer (Bruker Optik GmbH, Ettlingen, Germany).

### 2.4. UV–Vis Spectrophotometry

The concentrations of morin in liquid phase and the UV/Vis spectral characteristics of alkali lignin were determined on a DR 5000 UV/Vis Spectrophotometer (Hach Lange, Düsseldorf, Germany), supplied with 10 mm quartz cuvette cells. All spectra were recorded in the Vis region with a 2 nm slit width, 900 nm/min scan speed and very high smoothing. The standard curves of morin at pH = 1.2 (λ_max_ = 359 nm), pH = 6.8 (λ_max_ = 359 nm) and pH = 7.4 (λ_max_ = 394 nm) were characterized with a high degree of linearity with values of the correlation coefficients of R^2^ = 0.9976, 0.9989 and 0.9964, respectively.

### 2.5. In Vitro Release Studies

The in vitro release kinetics experiments were conducted by agitating morin-encapsulated lignin particle samples in 50 mL simulated enzyme-free gastrointestinal medium, which comprised PBS solution at pH = 1.2, 6.5 and 7.4 (pH was adjusted with 1 M HCl) at a temperature of 37 ± 0.5 °C in a digital water bath WNB 22 (Memmert GmbH, Büchenbach, Germany). Samples were taken at predetermined time intervals, and fresh medium of an equal volume was added to restore the total amount of the medium.

The concentration of released morin in the simulated physiological media was determined spectrophotometrically.

Volume corrections when processing the experimental data were performed via replacement of the drawn volume with simulated gastric fluid to avoid saturation of the remaining solution.

All experiments were carried out in triplicate, and the average values were taken to minimize random error. Blanks containing no biomolecules and replicates of each release point were used for each series of experiments.

### 2.6. Radical-Scavenging Activity

DPPH assay: The determination of DPPH scavenging by LP and LMP was adapted from a method previously described by Avelelas et al. (2019) [31]. Samples were prepared by mixing 3 mL of DPPH solution (0.1 mM in EtOH) and 200 µL of the microparticle suspensions. All samples were prepared in triplicate, protected from light and stirred. Absorbance at 517 nm was measured after 30 min. DPPH-scavenging activity (*DPPH*, %) was calculated as follows

(3) $$DPPH\;\left(\%\right)=\left(1-\frac{A_{x}}{A_{o}}\right)\cdot 100,$$

where *A_x_* is the absorbance of the samples and *A*_0_—the absorbance of the control.

ABTS assay: To generate ABTS^•+^, a cation radical solution was prepared based on the reaction of 7 mM ABTS solution in distilled water with 2.4 mM K_2_S_2_O_8_ in the dark for 24 h at 20 °C in a volumetric ratio of 1:1. Afterwards, *ABTS* was diluted with absolute EtOH to reach an absorbance of 0.7 measured at 734 nm. For the determination of the *ABTS*-scavenging capacity, 200 µL of sample was added to 3.6 mL of *ABTS* solution and the absorbance was measured spectrophotometrically at 734 nm. The trapping activity is reported as *ABTS* radical inhibition (%) [32]. The *ABTS*-scavenging capacity was calculated using Equation (4):

(4) $$ABTS\;\left(\%\right)=\left(1-\frac{I_{x}}{I_{o}}\right)\cdot 100$$

where *I_x_* is the absorbance of the control and *I*_0_—absorbance of the sample.

### 2.7. Mathematical Modelling, Statistical and Error Functions Analyses

The data obtained from the UV/Vis spectrophotometric, potentiometric titration and in vitro antioxidant activity studies were expressed as means ± standard deviations (SDs) from three repetitions. The statistical significance was determined by performing a Student’s t-test as the post-hoc test. A value of *p* < 0.05 was considered statistically significant. The in vitro release kinetics data were expressed as the average of three independent measurements, which were processed using the computer programs Origin 6.1 (OriginLab Corporation, Northampton, MA, USA) and Microsoft Excel 2010 (Microsoft Corporation, Redmond, WA, USA). The experimental release data were mathematically modelled using two release mathematical models: Korsmeyer–Peppas and the sigmoidal function model through non-linear regression analyses. The applicability of the mathematical models was interpreted on the basis of the mode of the experimental and model release curves, as well as of the values of the correlation coefficients (R^2^) and error functions (SSE, MSE, RMSE). The modelling approach was accomplished using XLSTAT statistical software for Excel (Microsoft Corporation, Redmond, WA, USA).

## 3. Results and Discussion

### 3.1. Physicochemical, Structural and Morphological Characterization of LPs and LMPs

The physicochemical, structural and morphological characteristics of alkali lignin, LPs and LMPs were determined based on particle size distribution, SEM, UV-Vis spectrophotometric, FTIR and potentiometric titration analyses.

**Figure 1:**
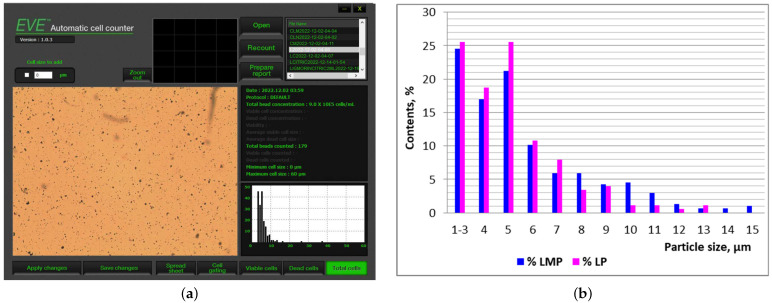
(**a**) Lignin microparticles EVE automatic beads counter image; (**b**) LP and LMP particle size distribution in 1 µL suspensions.

The total number of LMPs (306) in 1 µL of sample exceeded that of the LPs (179) by approximately 43%, which is an indication of the higher concentration of the flavonoid-loaded microparticles, denoting a higher extent of precipitation, probably due to the presence of the polyphenol. In addition, LMPs were characterized by a bigger average size (4.72 µm) as compared to LPs (4.57 µm). The latter observation is substantiated by the size distribution of both types of heterobiopolymer microparticles presented graphically in Figure 1, where the relative contents of the smaller LPs exceeded those of LMPs within the size range 1–7 µm, while a reverse tendency of distribution was established within the range 8–15 µm. The increased particle size of the morin-encapsulated lignin particles could be attributed to the spatial arrangement of the O-containing functional groups of lignin interacting with the phenolic groups of the encapsulated morin molecules leading to the formation of morin–monolignol units via a mechanism of radical coupling [33]. Similar results were reported by other authors [14].

**Figure 2:**
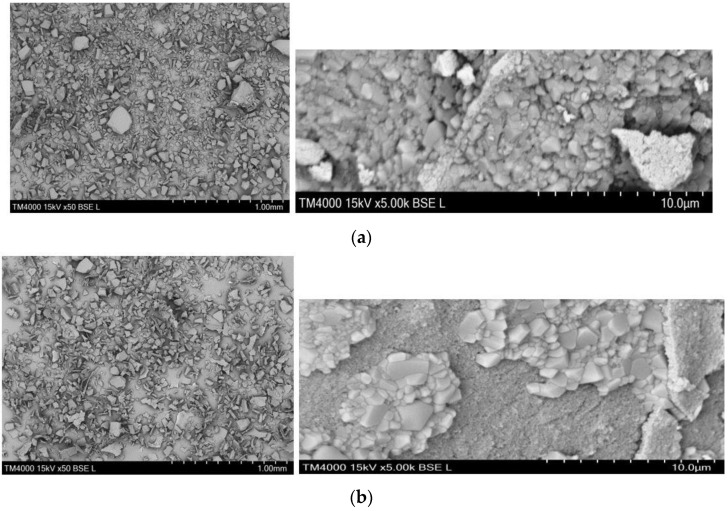
SEM images of (**a**) LP and (**b**) LMP at 50 and 5000 magnifications.

The SEM images of dried LPs and LMPs presented in Figure 2 show micro-sized lignin particles with a certain size variation. The micrographs in Figure 2a are in accordance with those reported in scientific literature [3,9]. Lu et al. (2012), Gomide et al. and Meng et al. presented similar images for lignin microparticles and characterized them as having an irregular morphology [4,9,34].

**Figure 3:**
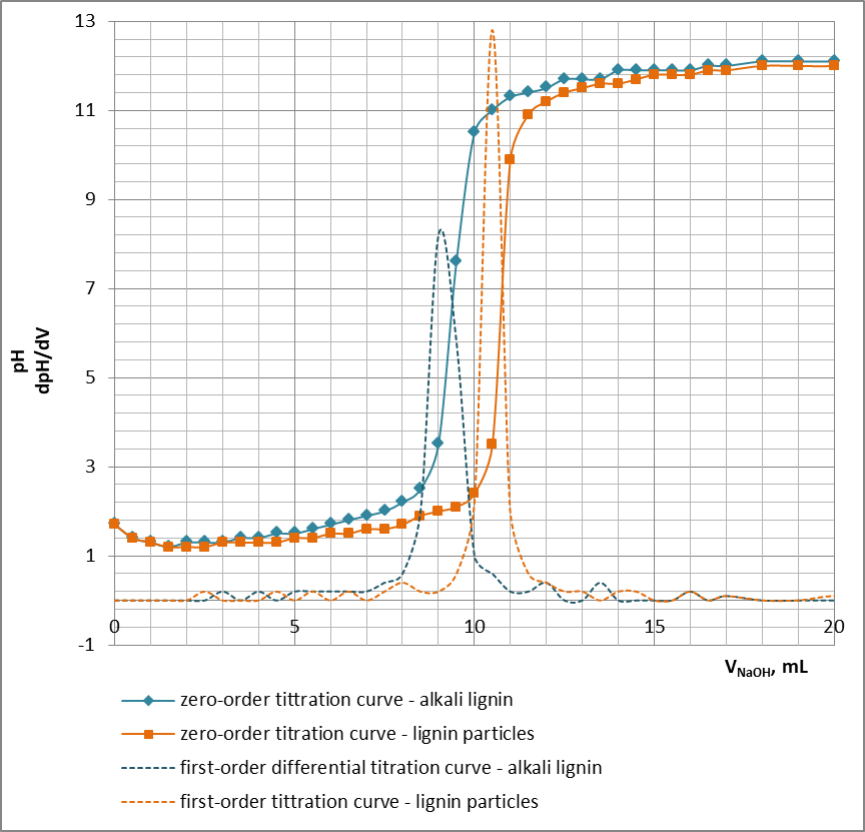
Zero-order and first-order differential potentiometric titration curves of alkali lignin and lignin microparticles.

Using potentiometric titration, the presence of a considerable number of acidic groups was determined in the pure heterobiopolymer (50 meq/g) and LP (55 meq/g) from the zero and first-order differential titration curves of alkali lignin and lignin microparticles (Figure 3). The greater number of acidic surface groups of lignin microparticles is probably a result of the presence of -NO_2_ groups due to probable reactions between the heteropolymer and HNO_3_.

**Figure 4:**
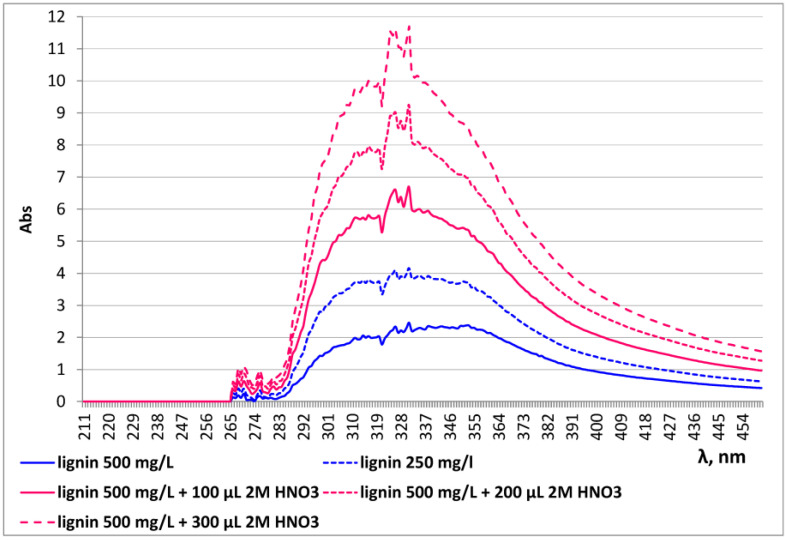
UV/Vis spectrophotometric spectra of aqueous alkali lignin solutions and HNO_3_-treated lignin solutions.

The UV/Vis absorption bands in the alkali lignin spectrum are assignable to various functional chemical groups: hydroxyl (Ar-OH phenolic, R-OH alcoholic), methoxy -OCH_3_, carbonyl -C=O, carboxyl -COOH [35]. As an aromatic heteropolymer, the distinctive absorption bands within the region 330–350 nm are characteristic of phenolic hydroxyl groups. According to literature data, the absorption maximum at around 350 nm is attributed to the presence of carbonyl groups conjugated with benzene rings [2]. To study the effect of the addition of HNO_3_ to alkali lignin as an antisolvent during the preparation of lignin microparticles on the physicochemical characteristics of the newly synthesized microparticles, we conducted UV/Vis spectrophotometric experiments with aqueous alkaline lignin solutions with two initial concentrations of 250 and 500 mg/L. Moreover, the absorption spectra of 500 mg/L lignin solutions containing 100, 200 and 300 μL 2N HNO_3_ were also measured (Figure 4). The latter differed from the usual absorption spectra of alkali lignin. The displacement of the absorption band at 350 nm and the increased intensity of the bands at 325–330 nm are apparently due to reactions of the heterobiopolymer with the acid. Similar results were reported by other scientific sources [2].

The encapsulation efficiency is the percentage of bioactive compound that is successfully entrapped into micro-/nano-particles. Encapsulation efficiency (*EE*%) was determined by means of the difference between the initial quantity of morin added during the synthesis procedure and spectrophotometrically determined quantity of the free non-entrapped morin in the filtrate divided but the initial flavonoid quantity. The encapsulation efficiency of morin by the lignin microparticles was 98.1%.

**Figure 5:**
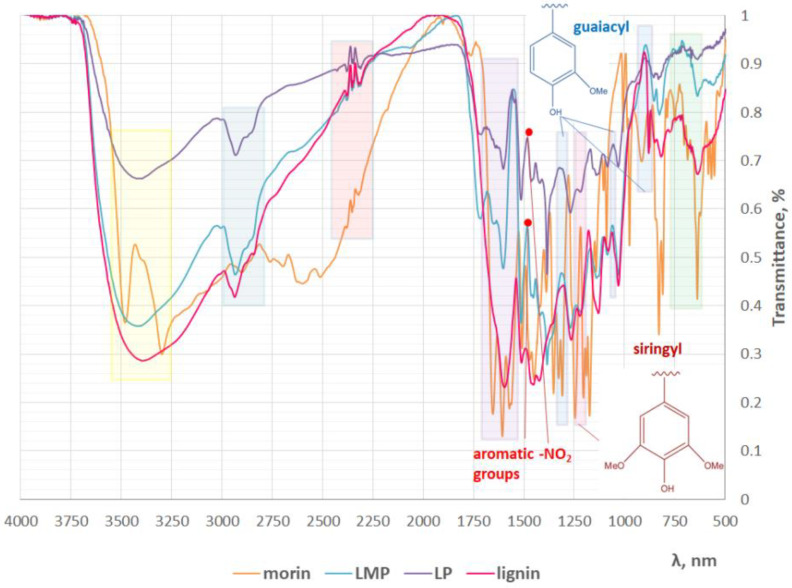
FTIR spectra of morin, alkali lignin, LPs and LMPs.

The FTIR spectra of morin, alkali lignin, blank lignin particles and morin-encapsulated lignin microparticles are illustrated in Figure 5. The assignments of the main FTIR peaks and bands and the corresponding wavelengths are presented in Table 1. The significantly higher peak intensity observed for alkali lignin at 3390 cm^−1^ assigned to O-H hydroxyl groups, when compared to that of LPs and LMPs and literature data of different types of pure lignins, is probably due to the introduction of -OH groups during alkali treatment [36]. The characteristic doublet at 3514 and 3300 cm^−1^ of the morin spectrum could be attributed to O–H stretching vibrations in the structure of flavonoid dimers. The infrared absorption spectra of LPs and LMPs were characterized by bands at 1477 cm^−1^, which can be assigned to aromatic -NO_2_ groups. The bands appearing at 1235–1220 cm^−1^ are related to syringyl structure bending, while those at 1300, 1188–1050 and 890 cm^−1^ can be attributed to the stretching of guaiacyl structures [1,9,23].

The infrared spectra of blank lignin particles and morin were merged to shape the spectrum of LMP. Even though some peaks shifted to higher or lower wavelengths, no noticeable new peaks were found in the spectra of LMP. Therefore, it could be supposed that morin was successfully encapsulated in LP without unexpected chemical reactions between the flavonoid and the heteropolymer.

### 3.2. In Vitro Release Study

**Figure 6:**
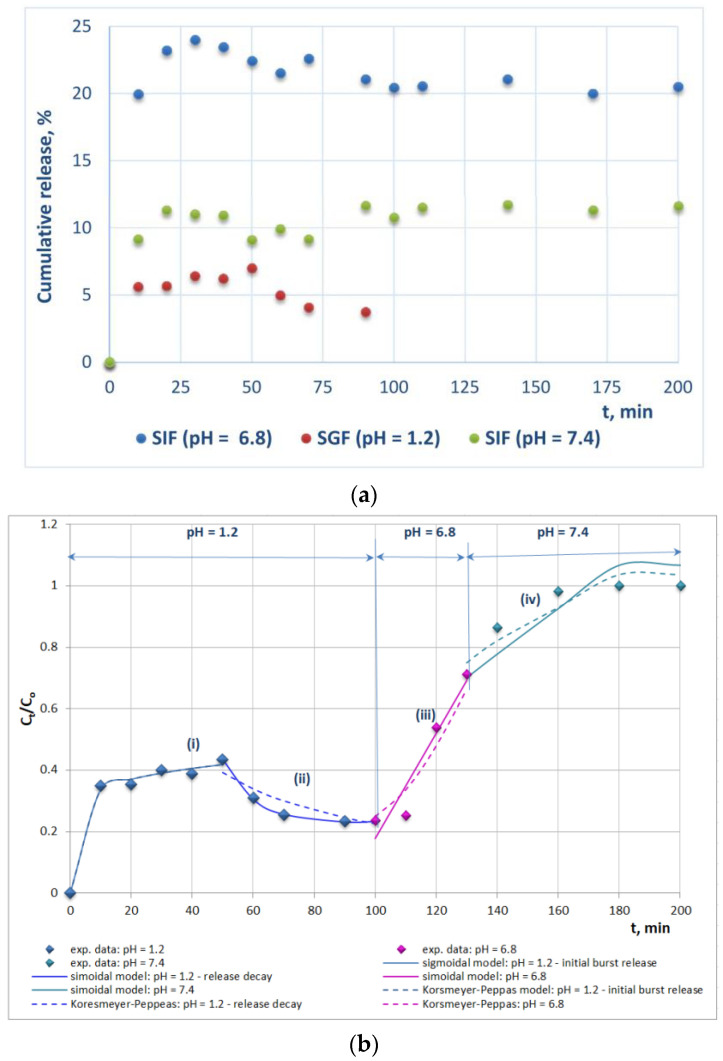
In vitro release kinetic curves of morin from LMPs: (**a**) experimental release kinetic curves in the three simulated physiological compartments; (**b**) experimental and model release profile throughout the simulated gastrointestinal tract—Korsmeyer–Peppas and sigmoidal model: (i) initial burst release in SGF; (ii) release decay in SGF; (iii) intensive release in SIF at pH = 6.8; (iv) slow and sustained release in the colon at pH = 7.4.

Unveiling the mechanism of the in vitro release of morin from the synthesized LMP could anticipate the behaviour of the flavonoid and the carrier in a real physiological medium and optimize the design of pharmaceutical formulations with improved bioavailability. There are several factors that influence the drug release rate. These factors include solubility of the drug, diffusion through the matrix, erosion of the matrix and desorption from the adsorbed drug, etc. [37]. According to pharmacokinetic studies using animal models, morin absorption occurs mainly in the small intestine and the colon [38,39,40]. However, the efficiency of in vitro and in vivo release of a polyphenol molecule from different delivery systems depends not only on the characteristics of the medium but also on the nature and the physicochemical properties of the carrier. Therefore, the in vitro release profile of morin-loaded lignin particles in three biorelevant media, simulated gastric fluid (SGF, pH = 1.2) and simulated intestinal fluid (SIF) at pH = 6.8 and pH = 7.4, were studied. The kinetic release curves are presented in Figure 6. The maximum cumulative release of morin reached in SGF was just 6.9% for 90 min. During the second stage, the microsystem was evaluated in simulated intestinal fluid (SIF, pH = 6.8), where the maximum cumulative release reached was 23%. The third stage of the pathway of a drug through the gastrointestinal tract is the colon, i.e., in SIF at pH = 7.4. The experimental results displayed 1.7 times lower release efficiency of the polyphenol as compared to that at pH = 6.8 and a two-fold higher extent of release than that achieved in SGF. The kinetic curves in Figure 6a present biphasic release patterns comprising a burst release in the initial period followed by a slow and sustained release in the three simulated physiological compartments. The release curve in Figure 6a, however, consists of four clearly defined regions: (i) initial burst release of the flavonoid up to 50 min in SGF, followed by (ii) a decay of the release efficiency during the next 50 min. In SIF at pH = 6.8, the curve displayed a steep slope indicative of (iii) intensive release of the natural compound within the time period 110–140 min and a subsequent stage of (iv) slow and sustained release in the colon at pH = 7.4 up to 200 min. The peculiarity of the mode of the second stage could be explained by the limited solubility of morin in strongly acidic medium, especially after the high initial rate of dissolution of flavonoid molecules, which were probably encapsulated on the surface of the lignin particles. In addition, at strongly acidic pH (pH = 1.2), both morin and lignin species exist in a protonated form, which is not favourable regarding morin solubility and consequently its release from the biopolymer carrier through diffusion [38]. Additionally, the acidic medium acts as an antisolvent for the lignin microparticles and probably increases their extent of crosslinking, thus “grasping” the encapsulated morin molecules more tightly.

An increase in pH leads to an increase in the net negative charge of lignin due to the intensified deprotonation of phenolic –OH groups, which in turn, results in increased concentrations of H^+^, reducing the pH on the surface of the heteropolymer [32]. Furthermore, as the pH was higher than the dissociation constant of the most acidic 2′–OH flavonoid group (pK_a1_ ≈ 3.5), morin molecules are also deprotonated. The partial ionization of the flavonoid favours its solubility, and the induction of repulsive forces with the negatively charged lignin surface triggers greater release efficiency during the last two stages of the process.

The semi-empirical model of Korsmeyer–Peppas is accepted as the most comprehensive mathematical equation applied for determination of the in vitro release profile of swellable polymers, which incited us to test its applicability with regards to our experimental results [41]. Moreover, the observed complex behaviour of the heteropolymer/flavonoid system requires the application of a modified mathematical methodology for its modelling. The sigmoidal model has been successfully applied for modelling the anomalous in vitro release behaviour of doxycycline from animal fodder [42], α-tocopherol-encapsulated zeolite microparticles [43] and catechin from clinoptilolite [44]. Thus, the applicability and suitability of this mathematical approach was also tested regarding the four stages of the current experimental release profile. The values of the model parameters and error functions determined via nonlinear regression analyses for both mathematical models are presented in Table 2.

The values of the exponential parameter in the Korsmeyer–Peppas model are indicative of the release mechanism. In this respect, for cylindrical particles, *n* = 0.45 corresponds to Fickian diffusion: the diffusion rate is lower than the relaxation rate; 0.45 < *n* < 0.89 indicates anomalous transport, i.e., intermediate state of commensurable rates of Fickian diffusion and relaxation; *n* = 0.89 means case II transport: independent of time release, regulated by macromolecular relaxation; and *n* > 0.89 designates super case II transport, revealing the contribution of diffusion, relaxation and erosion of the polymer chain [41]. Based on these assumptions and the data from Table 2, the release mechanism of morin from LMPs in SIF was found to be super case II transport due to the high values of *n* of 4.071 and 0.926 for stages (iii) and (iv), respectively. Probably, the release process is limited by diffusion, relaxation of the heteropolymer network due to swelling and partial dissolution leading to erosion.

The comparative assessment of the values of the experimental results, model data and error functions, as well as the modes of the experimental and model curves proved the relative applicability of the sigmoidal model. The values of the diffusional constant *k*_*s*1_, the relaxation constant *k*_*s*2_, the diffusional *k*_*s*1_ and the relaxation *n*_*s*2_ exponents enable the evaluation of the contributions of diffusion and/or relaxation mechanisms through the separate stages of the in vitro release process. Obviously the extent of contribution of both mechanisms is commensurable during the first stage of the release process in SGF due to the approximately equal values of the diffusional parameters with the relaxation constants. The values of *k*_*s*2_ for stages (iii) and (iv) were higher than these of *k*_*s*1_, which is indicative of the greater contribution of the relaxation mechanism over diffusion. The latter could be elucidated based on the susceptibility of lignin polymer chains to relaxation caused by mechanical stress (agitation) and the neutral-to-alkaline pH of the medium, which is a prerequisite for increased dissolution of the heteropolymer [45]. With respect to region (ii), the significantly higher value of the kinetic constant *k*_*s*2_ and the negative value of the exponent *n*_*s*2_ are denotative rather of shrinkage and compaction of the biopolymer matrix due to the low pH of the medium.

Consequently, based on the interpretative analyses of the modelling approach, it could be concluded that both applied mathematical models provoke similar findings outlining the general role of diffusion during the initial stages of the in vitro release process in SGF and the predominant contribution of relaxation and erosion in SIF.

The in vitro experimental results and the conclusions derived are therapeutically beneficial for clinical practice as they prove that due to the lower release efficiency of morin from LMPs in gastric medium, the newly synthesized microparticles are suitable for oral application as the risk of gastric irritation is lower as compared to that with the direct per os application of morin. In addition, LMPs offer a possibility of overcoming the limitations reported by other scientists associated with difficulties in administering high doses of morin orally resulting from the tendency of the flavonoid to form saturated solutions in the intestinal tract, thus hindering the drug dissolution process.

### 3.3. Antioxidant Activity and Radical-Scavenging Potential of Lignin Microparticles

DPPH and ABTS assays were applied for determination and comparative analyses of the radical-scavenging potential of LPs, LMPs, pure morin and alkali lignin.

**Scheme 1:**
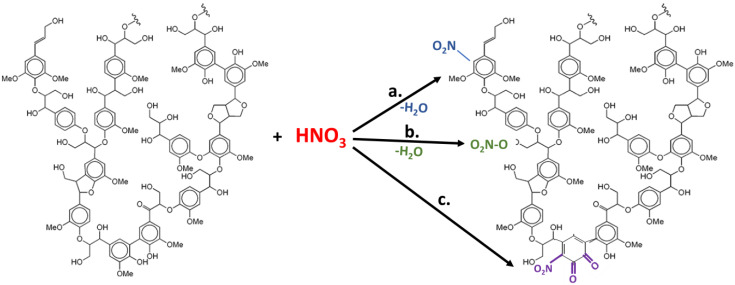
Chemical changes of lignin structure after modification with HNO_3_: (**a**) substitution reactions in the benzene ring; (**b**) esterification of aliphatic -OH groups; (**c**) oxidation of phenolic -OH and -OCH_3_ groups to the quinone structure.

**Figure 7:**
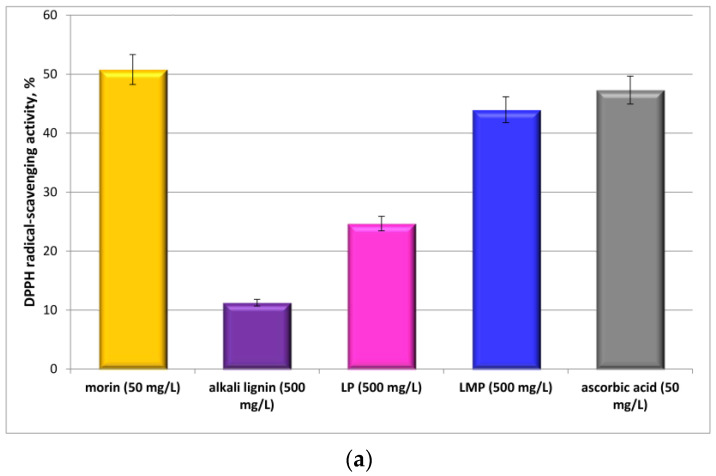

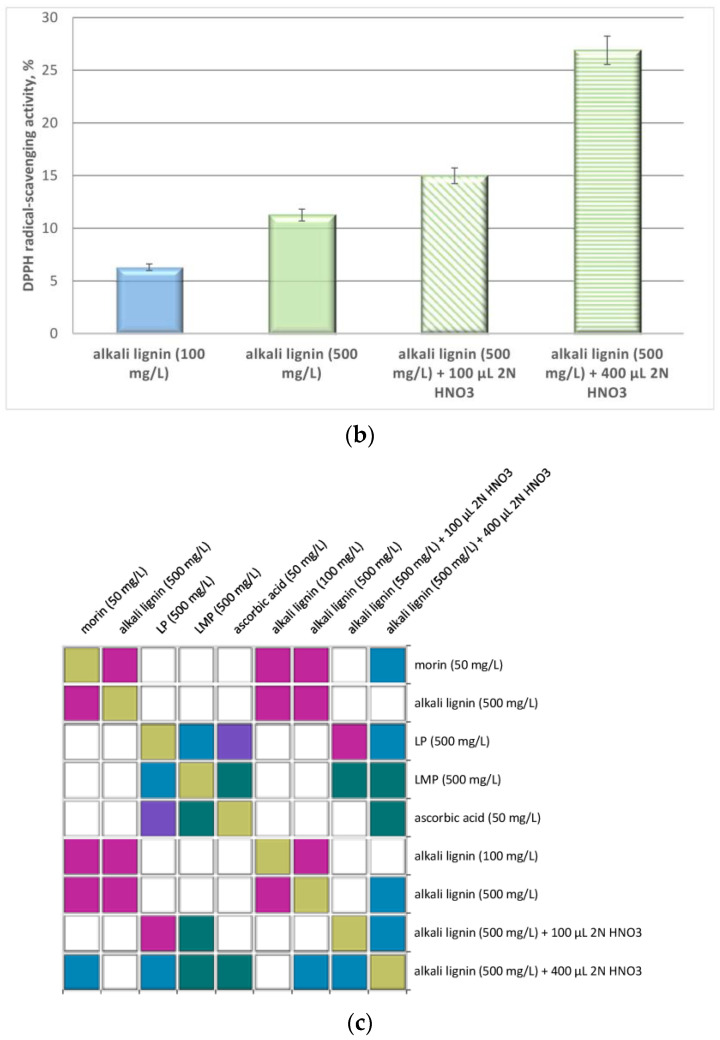
DPPH radical-scavenging activity, % (±SD). (**a**) DPPH activity of LPs, LMPs and pure substances. (**b**) Effect of nitric acid on the DPPH activity of alkali lignin. (**c**) *p*-values map: yellow—*p* = 0; purple—*p* < 0.05; violet—*p* < 0.08; turquoise—*p* < 0.15; blue—*p* < 0.3; white—*p* < 0.5.

The DPPH activity of the tested compounds and formulations is presented in Figure 7a. Alkali lignin exhibited lower antioxidant potential as compared to LPs with the same concentration. One of the possible explanations could be the fact that lignin macromolecules in aqueous solution are organized with the maximization of the number of intermolecular interactions between themselves and with the solvent molecules. Consequently, the aromatic hydroxyl groups of the heteropolymer are blocked and not easily accessed by the DPPH radical, while the pathway of the protons from the phenolic –OH groups on the surface of the microparticles is not restricted. Another possible reason for the increased scavenging potential of lignin particles could be possible modifications of the lignin structure as a result of the interaction with HNO_3_ (Scheme 1), which provoked the additional analyses of lignin solutions containing two doses of the acid (Figure 7b). DPPH activity increased directly with the increase in the acid dose. Considering the possible reaction products between lignin and nitric acid (Scheme 1c), it could be assumed that when each phenolic hydroxyl group scavenged more than one DPPH radical in the reaction system containing ethanol as a solvent, quinone groups may undergo a nucleophilic reaction with ethanol, resulting in the regeneration of the phenolic hydroxyl structure. Alternatively, after scavenging DPPH, the newly formed semiquinones may couple to form dimers. The latter multistage mechanism of proton transfer probably increases the scavenging activity of the free radicals. Similar results were reported by [45].

LMPs were characterized by higher DPPH-scavenging activity than LPs, while the antioxidant activity of morin surpassed that of LMPs and even of the positive control ascorbic acid. The probable reason for the observed high potential of the flavonoid is the large number of non-adjacent phenolic –OH groups that are capable of deprotonation. Although known as a universal antioxidant with proven radical-scavenging properties, ascorbic acid displayed lower activity probably due to the steric hindrance and the formation of intramolecular H-bonding between the adjacent phenolic hydroxyls. On the other hand, the lower DPPH potential of the LMP as compared to that of pure morin was probably a result of the blockage of certain Ar-OH groups due to intermolecular H-bonds and electrostatic interactions [33].

**Figure 8:**
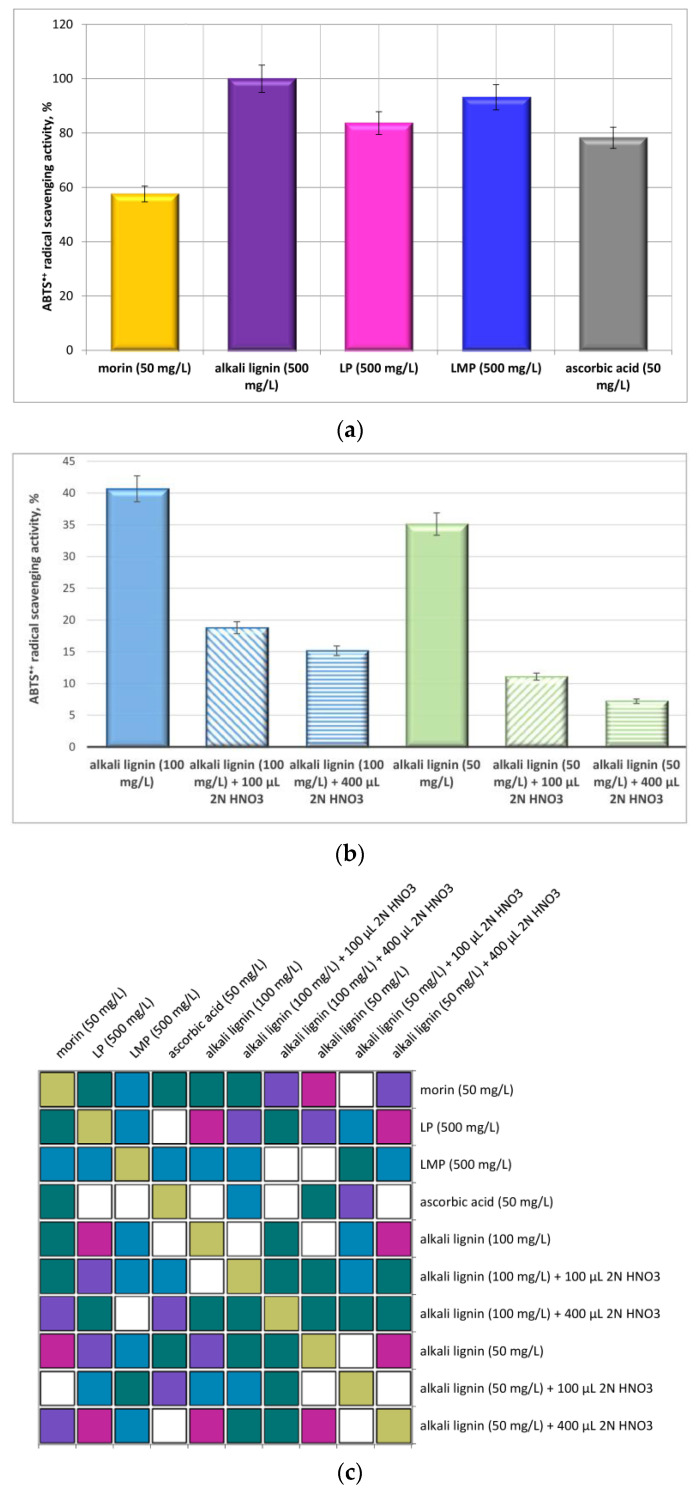
ABTS radical-scavenging activity, % (±SD). (**a**) ABTS activity of LPs, LMPs and pure substances. (**b**) Effect of nitric acid on the DPPH activity of alkali lignin. (**c**) *p*-values map: yellow—*p* = 0; purple—*p* < 0.05; violet—*p* < 0.08; turquoise—*p* < 0.15; blue—*p* < 0.3; white—*p* < 0.5.

The activity of LMPs, LPs, morin and alkali lignin toward the ABTS^●+^ radical was tested in ethanol as a medium, which does not favour deprotonation of the tested compounds (Figure 8). The experimental results demonstrated higher ABTS-scavenging activity of alkali lignin as compared to that of LPs (Figure 8a), which contradicts the DPPH assay results. Thus, additional experiments with lignin solutions containing two doses (100 and 400 µL) of HNO_3_ were accomplished (Figure 8b). The results outline a clear tendency of an inverse relationship between the acid quantity and the ABTS antioxidant activity of the mixtures.

The statistical significance of the experimental data of both assays was evaluated and is presented in Figure 7c and Figure 8c in the form of *p*-value maps.

**Figure 9:**
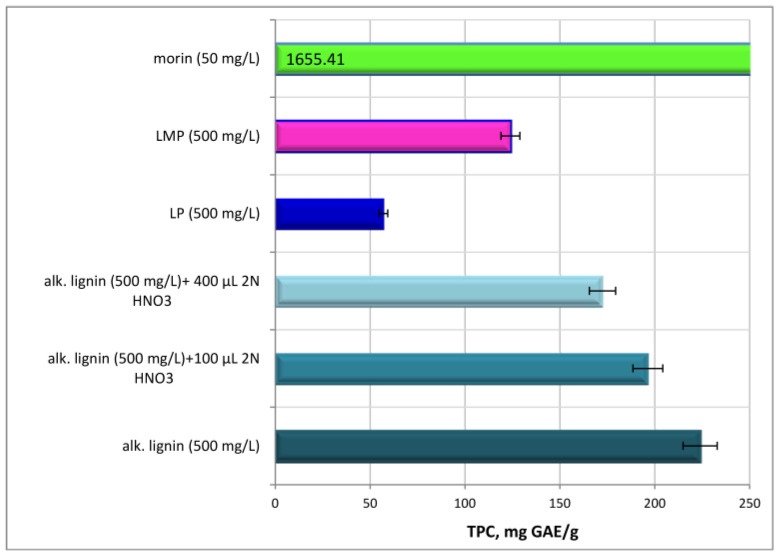
Total phenolic content (TPC) (mg GAE/g) (±SD) of LPs, LMPs and pure substances and effect of nitric acid on TPC of alkali lignin.

A similar tendency was established with regards to the values of TPC (Figure 9), indicating that increasing the quantity of the inorganic acid led to a decrease in the content of phenolic groups in the structure of alkali lignin. In addition, the higher value of TPC for LMPs, as compared to that with LPs is indicative of the enrichment of the heterobiopolymer particles with phenolic groups as a result of morin encapsulation.

From the viewpoint of the structural-activity concept electron-donating groups increase the electron cloud density of the benzene ring, which provokes a decrease in the dissociation energy of the phenolic O-H bond and consequently leads to enhancement of the free radical-scavenging ability [46]. Probably, the -NO_2_ groups in the structure of the nitric acid-treated alkali lignin (Scheme 1a,b) as strong electron-withdrawing groups enhance the dissociation energy of the phenolic O-H bond, thus lowering the antioxidant activity of the modified heteropolymer. In this respect, the fact that alkali lignin exhibited higher ABTS-scavenging ability than morin could be explained by the presence of methoxy groups at *ortho*- and/or *para*- positions on the benzene rings in the structure of lignin, which act as electron-donating substituents, although *ortho* –OCH_3_ substituents are also likely to cause steric effects [47].

The contradictory results of the order of antioxidant activity of morin, alkali lignin and LPs based on both applied assays are not obscure as similar conclusions were derived by a number of scientific investigations. According to the literature, the DPPH and ABTS assays are influenced by different structural properties, and the antioxidant behaviour depends on both the number and position of hydroxyl groups and other substituents of a molecule. According to Platzer et al. (2022) and Duan et al. (2022) the influence of additional methoxy groups could not be clarified conclusively. An increased number of Ar-OH and –OCH_3_ groups at the *ortho*- and *para*-position had a positive influence on the results of the DPPH and ABTS assays, as long as no steric hindrance occurred [48,49]. Non-etherified phenolic –OH groups, *ortho*-methoxy groups, hydroxyl groups and the double bond between the outermost carbon atoms in the side-chain contribute to the radical-scavenging ability of lignin [50].

In addition, the higher ABTS radical-scavenging activity of ascorbic acid as compared to that of morin indicated that an increase in the number of phenolic hydroxyl groups did not necessarily lead to higher antioxidant potential values.

Similar results were discussed by Adamcyk et al. (2021) and Ilyasov et al. (2020) who found that coumaric acids and isoferulic acid, inactive toward DPPH, were significantly active toward ABTS and even more active than certain diphenolic counterparts. The latter observations of our team and other authors lead to the conclusion that the observed deviations in the interpretation of the experimental results from the DPPH and ABTS assays may be attributed to the different mechanisms of the reactions of the antioxidants with ABTS, which are very complex and still not sufficiently clear [11,51].

The most significant conclusion, however, that was derived on the basis of the current study was the incontestably higher radical-scavenging potential of morin-loaded lignin microparticles as compared to that of LPs, which was substantiated by the two-fold higher phenolic content of LMPs as compared to that of LPs (Figure 9)—observations proven by the three applied assays. The DPPH activity of LMP was 1.76 times higher than that of LPs (Figure 7a), and the ABTS potential of LMPs surpassed that of LPs by approximately 10.2% (Figure 8a).

## 4. Conclusions and Future Perspectives

The in vitro experimental results and the conclusions derived are therapeutically beneficial for clinical practice as they prove that due to the lower release efficiency of morin from LMPs in gastric medium, the newly synthesized microparticles are suitable for per os application as the risk of gastric irritation is lower as compared to that with the direct oral administration of morin. The release mechanism of morin from LMPs was driven by Fickian diffusion and macromolecular relaxation. The novel morin-loaded lignin microparticles exhibited incontestably higher radical-scavenging potential than LPs according to DPPH and ABTS assays. The significance of the present results is expressed in the provision of assistance for the design of lignin-based drug-delivery formulations and/or functional foods based on their sustained in vitro release behaviour and enhanced antioxidant activity. The first reason for the proposed oral administration of the newly synthesized lignin–morin formulations is their micro scale, which is not suitable for injectable application. Additionally, the microparticles are intended to be applied as alternative supplements due to their valuable bioactivities.

The future investigations of the scientific team are inspired by the promising results obtained in the current research and are initiated by the urge for the development of “green” nanoscale lignin drug delivery formulations suitable not only for oral administration but also for injectable applications. Moreover, in vitro studies based on the verification of the safety, feasibility and efficiency of micro-/nano-scale lignin-based formulations using cell lines and in vivo clinical experiments are on their way.

## Figures and Tables

**Table 1:** FTIR spectra assignments of morin, alkali lignin, LPs and LMPs [22,23].

Wavenumber, cm^−1^	Alkali Lignin	LP	Morin	LMP
3700–3200	3390 phenolic hydroxyl groups (Ar-OH)	3408 O–H stretching band	3514, 3300 O–H stretching band; dimeric hydroxyl O–H stretching	3398 O–H stretching band
2950–2800	2935 C–H bond stretching	2929 C–H bond stretching	2898 C–H bond stretching	2906 C–H bond stretching
1700−1660	C=O bond vibrations of -COOH	C=O bond vibrations of -COOH	C=O bond vibrations of -COOH	C=O bond vibrations of -COOH
1660–1470	1595 aromatic ring vibrations and C–H, O–H, C=O bonds	1541 aromatic ring vibrations and C–H, O–H, C=O bonds	1659, 1629 -C=O stretching vibration; double bond alkenyl C=C stretching	1554 aromatic ring vibrations and C–H, O–H, C=O bonds
1500–1450	1479 C=C–C ring bonding; C–H bonds of -CH3 groups	1483 C=C–C ring bonding; C–H bonds of -CH3 groups 1477 aromatic -NO2 groups	1471 C=C–C ring bonding; C–H bonds of -CH3 groups	1481 C=C–C ring bonding; C–H bonds of -CH3 groups 1477 aromatic -NO2 groups
1350–1200	1301 guaiacyl ring and methoxy C–O stretching; O–H bonds of phenolic and non-ether groups 1234 syringyl units	1296 guaiacyl ring and methoxy C–O stretching; O–H bonds of phenolic and non-ether groups 1220 syringyl units	1307 -C−OH deformation vibrations 1245 -C-O-C bending	1305 guaiacyl ring and methoxy C–O stretching; O–H bonds of phenolic and non-ether groups 1234 syringyl units
1200–1100	1166 guaiacyl unit band 1160–1130 -OH bonds of secondary alcohols; condensed aromatic rings	1174 guaiacyl unit band 1160–1130 -OH bonds of secondary alcohols; condensed aromatic rings	1202; 1172 -C−OH stretching 1177 phenolic C–O stretching vibration	1188 guaiacyl unit band 1160–1130 -OH bonds of secondary alcohols; condensed aromatic rings
1100–1000	1058 band of guaiacyl unit; -OH bonds of primary alcohols	1047 band of guaiacyl unit; -OH bonds of primary alcohols	-	1062 band of guaiacyl unit; -OH bonds of primary alcohols
900–600	893 guaiacyl unit band	902 guaiacyl unit band	831 C–H vibration of aromatic ring 639 bending vibration of -OH alcoholic group	894 guaiacyl unit band

**Table 2:** Values of the model parameters, correlation coefficients and error functions of the applied in vitro release kinetics models.

In Vitro Release Stage	Model Parameters		Error Functions	
Korsmeyer–Peppas model				
(i)	*K_KP_*	0.249	R^2^	0.777
			SSE	0.001
	*n*	0.133	MSE	0.000
			RMSE	0.019
(ii)	*K_KP_*	8.737	R^2^	0.767
			SSE	0.007
	*n*	−0.793	MSE	0.002
			RMSE	0.042
(iii)	*K_KP_*	1.644 × 10^−9^	R^2^	0.933
			SSE	0.014
	*n*	4.071	MSE	0.007
			RMSE	0.084
(iv)	*K_KP_*	0.008	R^2^	0.835
			SSE	0.009
	*n*	0.926	MSE	0.004
			RMSE	0.066
Sigmoidal model				
(i)	*k*_*s*1_	0.120	R^2^	0.777
	*n*_*s*1_	0.132	SSE	0.001
	*k*_*s*2_	0.129	MSE	0.001
	*n*_*s*2_	0.134	RMSE	0.034
(ii)	*k*_*s*1_	0.034	R^2^	0.999
	*n*_*s*1_	0.400	SSE	2.457 × 10^−5^
	*k*_*s*2_	1.967 × 10^7^	MSE	0.012
	*n*_*s*2_	−4.037	RMSE	0.109
(iii)	*k*_*s*1_	0.213	R^2^	0.916
	*n*_*s*1_	0.697	SSE	0.013
	*k*_*s*2_	0.860	MSE	0.014
	*n*_*s*2_	0.386	RMSE	0.111
(iv)	*k*_*s*1_	0.114	R^2^	0.785
	*n*_*s*1_	0.523	SSE	0.038
	*k*_*s*2_	0.768	MSE	0.019
	*n*_*s*2_	−0.010	RMSE	0.138

## Data Availability

The data presented in this study are available on request from the corresponding author.
